# Organ-specific role of neutrophil extracellular traps in shaping the postoperative thrombo-inflammatory niche in pancreatic ductal adenocarcinoma: a multi-model machine learning and mechanistic study

**DOI:** 10.3389/fimmu.2025.1728391

**Published:** 2025-12-09

**Authors:** Songping Cui, Di Wang, Shaocheng Lyu, Jincan Huang, Ren Lang, Jing Wang

**Affiliations:** 1 Department of Thoracic Surgery, Beijing Institute of Respiratory Medicine and Beijing Chao-Yang Hospital, Capital Medical University, Beijing, China; 2 Department of Liver Transplantation, First Central Hospital of Tianjin Medical University, Tianjin, China; 3 Department of Hepatobiliary and Pancreaticosplenic Surgery, Beijing Chaoyang Hospital, Capital Medical University, Beijing, China; 4 Mass General Cancer Center, Massachusetts General Hospital, Harvard Medical School, Boston, MA, United States

**Keywords:** neutrophil extracellular traps, portal vein thrombosis, pancreatic ductal adenocarcinoma, prognosis, explainable artificial intelligence

## Abstract

**Background:**

Neutrophil extracellular traps (NETs) play a crucial role in thrombosis formation and tumor progression. However, the association between NETs and portal vein thrombosis (PVT) is not well understood.

**Methods:**

We retrospectively and prospectively developed and validated a predictive model for postoperative PVT following radical surgery for pancreatic ductal adenocarcinoma (PDAC) from Jan. 2011 to Dec. 2022 and Jan. 2023 to Dec. 2023. 30 clinicopathological variables, including the novel biomarker NETs, were evaluated. 11 ML algorithms were compared based on several evaluation indexes including AUC. SHapley Additive exPlanations (SHAP) analysis was utilized for feature ranking and interpretability.

**Results:**

In the retrospective study, a total of 306 patients were included, among which 70 (22.9%) developed PVT. In the prospective study, a total of 64 patients were enrolled and the incidence of PVT was 25%. eXtreme gradient boosting (XGBoost) demonstrated superior discriminative performance among 11 evaluated ML-models. After feature reduction based on ranked feature importance, a final interpretable XGBoost model comprising 7 variables was established. This model accurately predicted postoperative PVT in both training (AUC = 0.906) and validation (AUC = 0.823) cohorts, and has been translated into a convenient tool to facilitate its utility in clinical settings.

**Conclusion:**

In patients with PDAC, higher NETs levels are correlated with an increased risk of developing PVT and are indicative of poor prognosis. Assessment of Cit-H3 and D-dimer levels may aid clinicians in predicting PVT.

## 1 Introduction

Neutrophils are the most abundant group of white blood cells in human peripheral blood and are an important component of the immune system (1, 2). When microorganisms invade the human body, neutrophils defend against them by engulfing the pathogens and secreting antibacterial substances from their cytoplasmic granules. Additionally, neutrophils can be activated through various mechanisms in response to different stimuli, leading to the formation of neutrophil extracellular traps (NETs) (3, 4). NETs can promote tumorigenesis, progression, and the occurrence of cancer-associated thrombosis (CAT) and other related pathological processes (5–8).

Portal vein thrombosis (PVT) is defined as the formation of a blood clot in the portal vein or its branches, with or without extension into the superior mesenteric vein (SMV) or splenic vein (SV) (9). PVT is one of the common postoperative complications in patients with pancreatic ductal adenocarcinoma (PDAC), especially in borderline resectable pancreatic cancer (10, 11). Patients of this type frequently have tumors that invade the portal venous system (PVS), necessitating portal venous resection (PVR) and various forms of reconstruction during surgery (12, 13). PVR, vascular graft implantation, diabetes, etc., are related factors for the occurrence of PVT after surgery (10, 11, 14).

The pathophysiology of venous thromboembolism (VTE) has long emphasized a series of factors that constitute Virchow’s triad: hypercoagulability, endothelial injury, and blood stasis. NETs play a crucial role in the formation of VTE and are closely interconnected with the various components of Virchow’s triad. NETs increase thrombin generation by binding with tissue factor (TF), procoagulant extracellular vesicles, and TF-containing extracellular vesicles (15–17). NETs damage the endothelial cells’ glycocalyx and increase endothelial permeability, which may further contribute to dysregulated inflammation, impaired microcirculatory blood flow, tissue hypoperfusion (18, 19). NETs’ histone-DNA scaffold provides a new adhesion platform for platelets, promoting their activation and aggregation, and also adds an extra framework for the fibrin network to stabilize the thrombus (20, 21).

Currently, there are still relatively few studies on NETs and PVT. Therefore, we designed this study to investigate the relationship between NETs, PVT, aiming to provide a reference for the clinical prevention, treatment, and mechanism exploration of PVT, thereby improving the prognosis of PDAC patients.

## 2 Materials and methods

### 2.1 Study population

#### 2.1.1 Retrospective study

We retrospectively collected data from patients who underwent radical pancreatic cancer surgery at our hospital between January 2011 and December 2022.

The inclusion criteria are as follows: (1) age over 18 years; (2) pathologically confirmed PDAC; (3) absence of distant metastasis; (4) no history of neoadjuvant therapy prior to surgery; (5) preoperative examination showed no obvious PVT. The exclusion criteria are as follows: (1) pregnancy or breastfeeding; (2) simultaneous malignant tumors in other systems; (3) current blood system disease; (4) insufficient follow-up information.

#### 2.1.2 Prospective study

We prospectively collected data from patients who underwent radical pancreatic cancer surgery at our hospital from January 2023 to December 2023.

The inclusion criteria are as follows: (1) age 18 to 75, no gender restriction; (2) preoperative evaluation indicated no contraindication of surgery; (3) preoperative estimated survival time exceed 12 months; (4) patients and their family members agreed with the protocol and signed informed consent form.

The exclusion criteria are as follows: (1) preoperative examination revealed the presence of preoperative PVT; (2) detected distant metastasis during surgery and gave up radical operation intraoperatively; (3) insufficient follow-up information.

All patients were informed about the treatment procedure and signed consent forms. For patients requiring vascular grafts, all implants were allogeneic vessels. The allogeneic vessels were obtained by our hospital’s Organ Procurement Organization during organ donation. All patients were from the same center and the same surgical team, adhering to identical inclusion/exclusion criteria, perioperative management pathways, and anticoagulation prophylaxis protocols. All patients received prophylactic anticoagulation with low molecular weight heparin immediately after surgery when there is no significant risk of bleeding, and switch to aspirin 100 mg QD until 6 months after surgery.

### 2.2 Clinical data collection

We gathered data from the electronic medical record system, which included patient demographics (age, sex), underlying health conditions (diabetes, coronary heart disease, hypertension), laboratory results (neutrophil count, platelet count, total bilirubin, serum albumin, D-dimer), details of the surgery (approach, duration, bleeding volume, transfusion volume), pathological findings (type, differentiation grade, TNM staging), and the presence of PVT.

All PVT events in this study were diagnosed based on contrast-enhanced CT imaging of the portal venous system. The radiological criterion was the presence of a definite low-density filling defect within the lumen of the portal vein, superior mesenteric vein, or splenic vein. Thromboses caused by direct tumor invasion or local recurrence were excluded.

Follow-ups were conducted in the 1st and 3rd months postoperatively, every 3 months for the first year, and every 6 months thereafter. The follow-up examinations mainly included laboratory tests and enhanced abdominal CT scans. All patients were regularly followed up postoperatively, with records kept of PVT occurrence. The last follow-up was in December 2024.

### 2.3 Detection and quantification of biomarkers

This study included tissue samples from 370 patients. For each patient, three types of samples were systematically collected: tumor tissue, adjacent tissue (2 cm from the tumor margin), and nearby normal tissue. To ensure sample representativeness and address tumor heterogeneity, at least three independent paraffin blocks were prepared for each tissue type per patient, with 3–5 consecutive sections from each block used for subsequent immunofluorescence staining and quantitative analysis. After obtaining the tumor and adjacent tissues during surgery, we immediately placed them in saline or PBS for initial processing. The tissues were then fixed in 10% formalin for 12–24 hours, followed by dehydration with a gradient of ethanol, clarification with xylene, and infiltration with paraffin. The tissues were embedded in paraffin blocks and sectioned into 4-6μm thick slices. We used immunofluorescence to detect the expression of NETs in PDAC tissues. Staining was performed with anti-myeloperoxidase (MPO) antibody (22225-1-AP, Proteintech, dilution 1:50) and anti-citrullinated histone H3 (Cit-H3) antibody (ab5103, Abcam, dilution 1:3000). For fluorescence detection, we used the opal 3-plex manual detection kit (NEL811001KT, Akoya). We performed dual immunofluorescence staining for MPO and Cit-H3 on tumor tissue sections. Two independent pathologists counted MPO^+^Cit-H3^+^ double-positive cells in four randomly selected high-power fields (200×) per sample, and the counts were averaged. The NET% was defined as the proportion of MPO+Cit-H3+ double-positive cells, reflecting the extent of NET formation in the tumor microenvironment. Patients were stratified into high- and low-NET groups based on the median NET% value. We detected biomarkers such as Cit-H3, neutrophil elastase (NE), and P-selectin in human peripheral blood using the ELISA method (22).

### 2.4 Feature selection

To prevent model overfitting and enhance predictive performance, all variables were first subjected to feature selection. The Least Absolute Shrinkage and Selection Operator (LASSO) regression was applied for preliminary variable screening. By imposing an L1 regularization penalty on the regression coefficients, LASSO shrinks some of them to zero, thereby achieving simultaneous variable selection and model simplification. In this study, the optimal penalty parameter (λ) was determined through 10-fold cross-validation, and variables with non-zero coefficients corresponding to this λ were retained as candidate features.

To further verify the robustness of the selected features, Recursive Feature Elimination (RFE) was subsequently employed. RFE iteratively constructs models, evaluates feature importance, and eliminates the least informative variables in successive steps, ultimately identifying the optimal subset of predictors. Similarly, 10-fold cross-validation was used to assess model performance across different feature subset sizes and to determine the most predictive combination of variables.

Finally, variables identified by both LASSO regression and RFE were extracted as the final input features for subsequent machine-learning model construction and validation.

### 2.5 Model development and explanation

A total of 11 machine learning (ML) algorithms-adaptive boosting (AdaBoost), artificial neural network (ANN), decision tree (DT), extra trees (ET), gradient boosting machine (GBM), k-nearest neighbors (KNN), Light Gradient Boosting Machine (LightGBM), logistic regression (LR), random forest (RF), support vector machine (SVM), and extreme gradient boosting (XGBoost)-were implemented to predict postoperative portal vein thrombosis (PVT). Hyperparameter optimization was performed using a randomized search framework (Optuna) integrated with manual fine-tuning to achieve optimal model configurations. Model development was carried out under a 10-fold cross-validation scheme within the training cohort to ensure robustness and minimize overfitting. The predictive performance and model reliability were comprehensively evaluated using standard diagnostic metrics, including the area under the receiver operating characteristic curve (AUC), sensitivity, specificity, positive predictive value (PPV), negative predictive value (NPV), accuracy, Brier score, and F1 score. External validation was conducted in an independent prospective testing cohort to assess model generalizability across populations. To enhance interpretability, SHapley Additive exPlanations (SHAP) were employed to quantify the contribution of each input feature to the model’s predictions. SHAP analysis provided both global and local interpretability: globally, it offered consistent and unbiased estimates of feature importance, elucidating their relationships with postoperative thrombotic events; locally, it enabled patient-specific interpretive insights by explaining individualized prediction outputs.

Finally, probability calibration was performed using Platt scaling, thereby improving the clinical reliability of probability estimates and reducing potential risks of model overconfidence or decision-making bias in real-world applications.

### 2.6 Webpage deployment tool based on streamlit framework

For practical clinical deployment, the optimized prediction model was implemented into a user-friendly web-based application using the Streamlit Python framework. Upon entry of individual patient features, the platform generates the probability of PVT occurrence for personalized decision support.

### 2.7 Statistical analysis

Data analyses were conducted using Python version 3.9.13 (https://www.python.org) and SPSS Statistical Software Version 23.0 and R version 4.1.0 (https://www.r-project.org). Continuous variables with skewed distributions were presented as median with interquartile range and compared using the Mann-Whitney U test or Kruskal-Wallis H test. Cate- gorical variables were presented as numbers with per- centages and compared using the Chi-square test or Fisher’s exact test. The analysis of covariance (ANCOVA) was used to adjust for the confounder. The AUCs were used to evaluate the predictive power, and the optimal cutoff value was established by maximizing the Youden index. A two-tailed P value < 0.05 was considered statistically significant.

## 3 Results

### 3.1 Retrospective study

#### 3.1.1 Basic perioperative information

**Figure 1:**
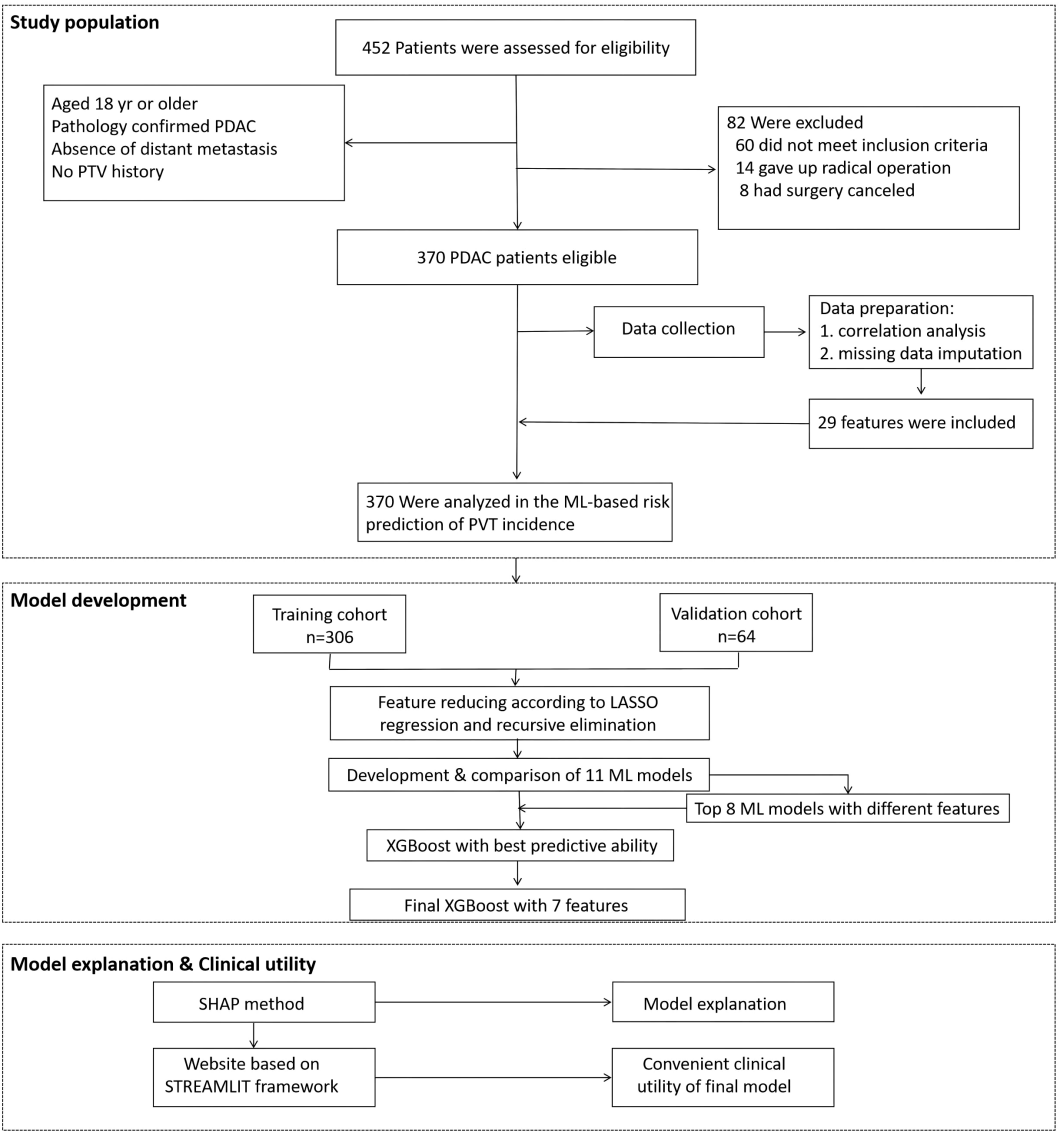
Flow chart of the study design.

In the retrospective study, a total of 306 patients were included, of which 70 had PVT, with an incidence rate of 22.9%, and 236 were non-PVT. The average age is 65.00 (58.00, 71.00) years, including 128 females (41.8%). The demographic and clinical characteristics of all patients are detailed in **Table 1**. Details of the study design are displayed in **Figure 1**.

**Table 1:** Clinicopathologic features and operative characteristics.

Variables	Training set		P Value	Test set		P Value
	Non-PVT (n=236)	PVT (n=70)		Non-PVT (n=48)	PVT (n=16)	
Age (y)^b^	64.00 (57.00, 71.00)	66.00 (63.00, 71.75)	0.032	59.50 (55.00, 66.00)	66.50 (63.50, 69.25)	0.015
Female sex^a^	99 (41.95%)	29 (41.43%)	0.989	26 (54.17%)	6 (37.50%)	0.386
Underlying comorbidity^a^						
Hypertension	98 (41.53%)	20 (28.57%)	0.069	19 (39.58%)	6 (37.50%)	0.978
DM	73 (30.93%)	27 (38.57%)	0.293	16 (33.33%)	3 (18.75%)	0.353
CHD	30 (12.71%)	5 (7.14%)	0.284	9 (18.75%)	2 (12.50%)	0.716
History of abdominal	13 (5.51%)	5 (7.14%)	0.318	3 (6.25%)	1 (6.25%)	0.999
Smoking	77 (32.63%)	28 (40.00%)		12 (25.00%)	2 (12.50%)	0.487
Surgical approach^a^			<0.001			0.005
Non-PVR	138 (17.28%)	24 (16.90%)		33 (68.75%)	5 (31.25%)	
End-to-end anastomosis	78 (32.30%)	27 (25.35%)		13 (27.08%)	6 (37.50%)	
Vascular replacement	20 (14.61%)	19 (21.13%)		2 2 (4.17%)	5 (31.25%)	
Operation time (h)^b^	9.00 (8.00, 12.00)	10.00 (9.00, 13.00)	0.005	9.00 (8.00, 12.00)	10.00 (9.00, 11.25)	0.229
Hemorrhage (mL)^b^	500.00 (400.00, 800.00)	600.00 (400.00, 975.00)	0.018	500.00 (400.00, 800.00)	550.00 (400.00, 800.00)	0.383
Transfusion volume (ml)^a^			0.038			0.155
0	131 (55.51%)	27 (38.57%)		34 (70.83%)	9 (56.25%)	
≥400	44 (18.64%)	16 (22.86%)		5 (10.42%)	5 (31.25%)	
≥800	61 (25.85%)	27 (38.57%)		9 (18.75%)	2 (12.50%)	
NETs (%)^b^	0.08 (0.04, 0.23)	0.25 (0.14, 0.55)	<0.001	0.13 (0.08, 0.20)	0.23 (0.06, 0.33)	0.195
TNM^a^			0.061			0.309
I	65 (27.54%)	10 (14.29%)		12 (25.00%)	2 (12.50%)	
II	108 (45.76%)	35 (50.00%)		25 (52.08%)	7 (43.75%)	
III	63 (26.69%)	25 (35.71%)		11 (22.92%)	7 (43.75%)	
Differentiation^a^			0.077			0.526
Poorly	71 (30.08%)	30 (42.86%)		16 (33.33%)	4 (25.00%)	
Moderately	146 (61.86%)	38 (54.29%)		29 (60.42%)	12 (75.00%)	
Highly	19 (8.05%)	2 (2.86%)		3 (6.25%)	0 (0.00%)	
Chemotherapy, only^a^	138 (58.47%)	35 (50.00%)	0.263	28 (58.33%)	12 (75.00%)	0.372
Laboratory values^b^						
WBC (x 10^9^/L)	5.75 (4.60, 6.90)	5.85 (4.90, 7.57)	0.319	5.85 (4.77, 6.67)	5.40 (4.40, 6.45)	0.558
Neutrophil (x 10^9^/L)	3.60 (2.70, 4.60)	3.70 (3.00, 4.97)	0.151	3.51 (2.88, 4.36)	3.64 (2.99, 4.36)	0.648
PLT (x 10^9^/L)	204.00 (165.00, 255.25)	217.00 (170.50, 268.00)	0.170	199.00 (153.75, 246.75)	185.50 (146.25, 277.75)	0.936
Hemoglobin (g/dL)	124.00 (113.00, 134.00)	119.50 (111.25, 137.50)	0.859	120.50 (113.00, 139.25)	129.50 (114.75, 139.00)	0.679
Albumin (g /L)	37.85 (34.15, 40.82)	38.05 (34.47, 42.35)	0.515	36.85 (32.90, 39.90)	38.35 (32.68, 40.02)	0.792
Total bilirubin (umol /L)	19.55 (10.20, 123.53)	36.80 (11.22, 149.25)	0.287	23.95 (11.52, 101.70)	16.45 (12.60, 78.50)	0.664
Pre-D-dimer (ug/mL)	0.41 (0.25, 0.57)	0.52 (0.39, 0.76)	0.039	0.37 (0.23, 0.53)	0.66 (0.50, 0.91)	<0.001
D-dimer(D3) (ug/mL)	2.51 (2.08, 3.76)	3.97 (3.12, 4.84)	<0.001	2.40 (1.81, 3.11)	3.95 (2.53, 4.82)	0.006
ALT ((U/L)	32.00 (16.00, 92.50)	48.00 (20.25, 117.00)	0.106	31.00 (15.75, 60.25)	49.00 (15.00, 137.50)	0.281
AST ((U/L)	31.00 (18.00, 65.00)	41.50 (21.25, 97.50)	0.041	23.50 (16.00, 49.50)	56.50 (24.75, 102.00)	0.057
GGT (U/L)	107.00 (21.00, 430.50)	199.00 (29.25, 472.50)	0.139	135.00 (17.75, 299.75)	31.00 (18.50, 183.00)	0.721
CA19-9 (U /mL)	175.05 (38.90, 494.15)	420.45 (40.58, 1297.18)	0.011	87.50 (9.40, 280.00)	466.15 (148.33, 1676.60)	0.003
CEA (ng /mL)	2.10 (1.30, 4.15)	3.10 (1.65, 4.97)	0.078	3.15 (1.90, 4.32)	4.75 (3.25, 7.43)	0.052

PVT, Portal vein thrombosis; DM, Diabetes Mellitus; CHD, Coronary Heart Disease; PVR, portal venous resection; NETs, neutrophil extracellular traps; WBC,White Blood Cell; PLT, Platelet Count; ALT, Alanine aminotransferase; AST, Aspartate aminotransferase; GGT, Gamma-glutamyl transferase; CA19-9, Carbohydrate antigen 19-9; CEA, Carcinoembryonic antigen. ^a^Number (%); ^b^Median (range).

#### 3.1.2 Experimental validation of the role of NETs in the development of PVT

**Figure 2:**
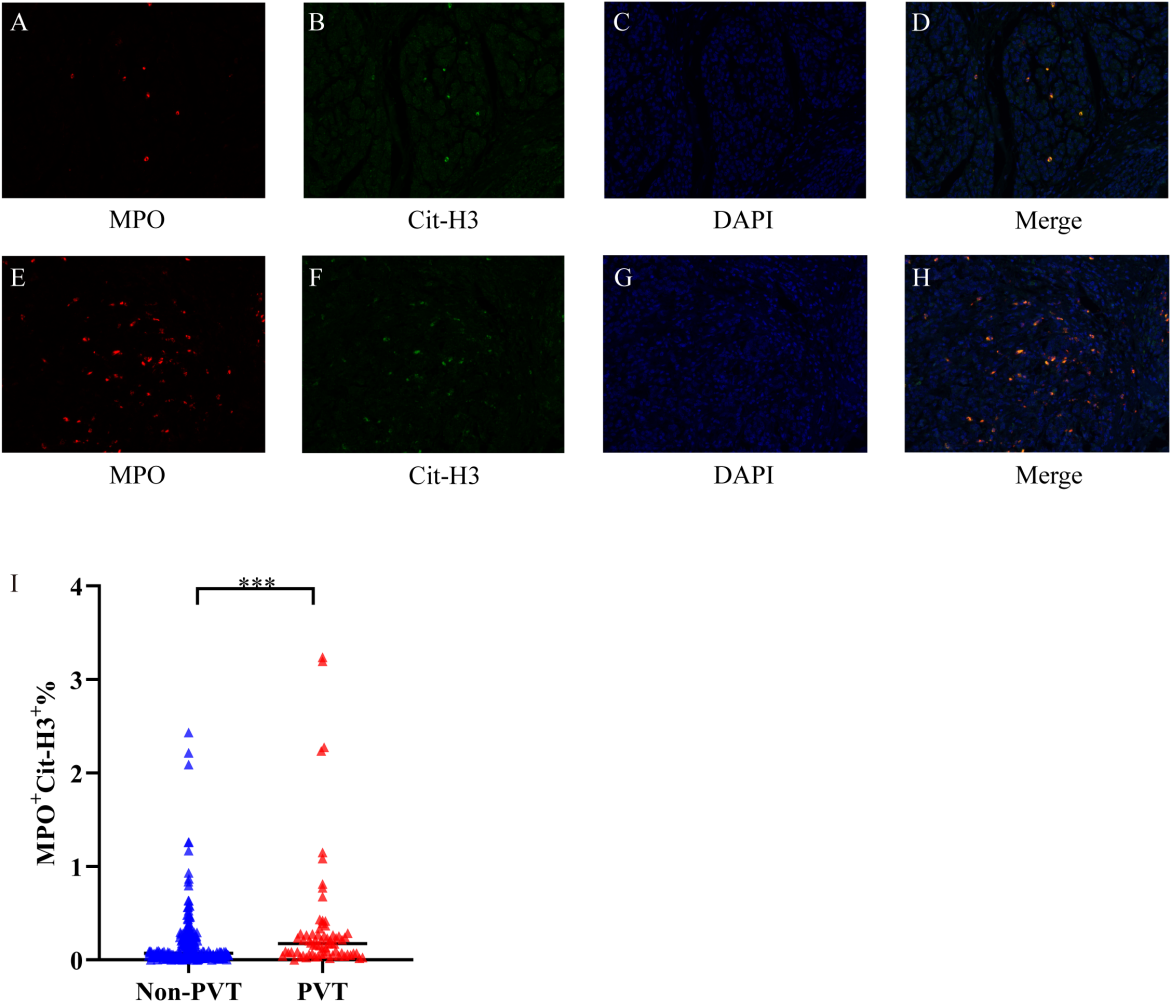
Differences in the expression of NETs in tumor tissues among different PDAC patients. **(A–D)** Immunofluorescence images of tumor tissues in the low NETs group showing MPO, Cit-H3, DAPI, and their merged images, respectively. **(E–H)** Immunofluorescence images of tumor tissues in the high NETs group showing MPO, Cit-H3, DAPI, and their merged images, respectively. **(I)** The difference in MPO+Cit-H3+% expression between the Non-PVT group and the PVT group. *** indicates P<0.001.

We used MPO and Cit-H3 antibodies to label NETs in tumor tissues and classified them into low NETs and high NETs groups based on the proportion of MPO+Cit-H3+ cells (**Figures 2A–H**). Through intergroup comparison, we found that the proportion of MPO+Cit-H3+ cells in the PVT group was significantly higher than that in the Non-PVT group, and demonstrated a statistically significant difference(P<0.05) (**Figure 2I**).

#### 3.1.3 Model selection and comparative performance

**Figure 3:**
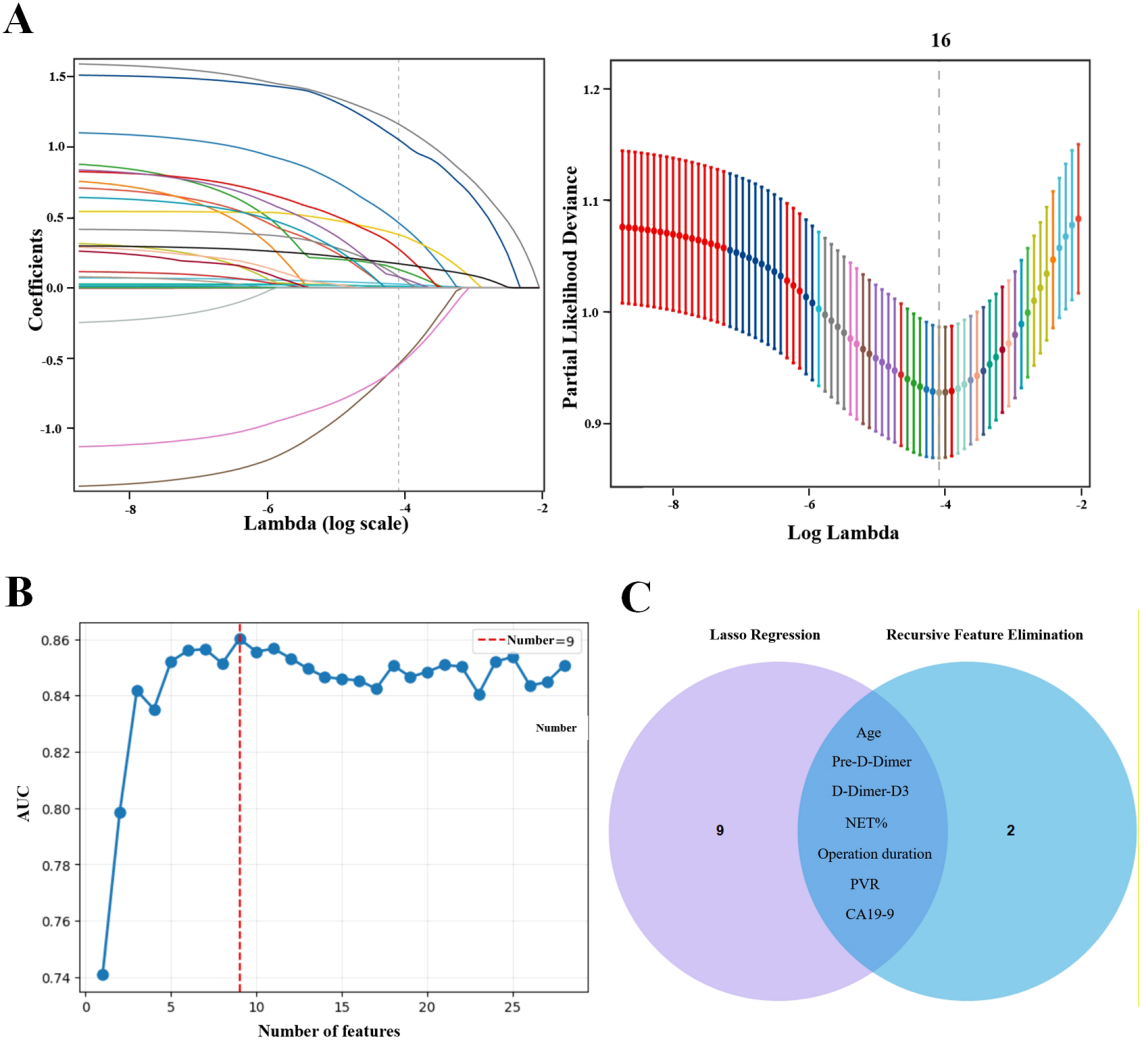
Feature selection. **(A)** Coefficient path of the LASSO regression. The plot illustrates how the coefficients of each predictive variable progressively shrink toward zero as the regularization parameter λ (log scale) increases. **(B)** Feature selection curve showing model performance across varying feature counts. The vertical dashed line denotes the optimal number of features corresponding to the highest AUC. **(C)** Venn diagram displaying the intersection of variables identified by LASSO regression and recursive feature elimination (RFE).

A total of 29 clinical and laboratory variables were included in this study. To prevent overfitting and optimize feature dimensionality, an initial screening was conducted using the LASSO regression model, which identified 16 non-zero coefficient variables (**Figure 3A**). Subsequently, to further refine the feature set and eliminate redundant information, RFE was employed to iteratively evaluate model performance and rank variable importance. The RFE analysis yielded nine key predictive features (**Figure 3B**).

To ensure the robustness and stability of feature selection, the intersection of variables identified by LASSO and RFE was taken, resulting in seven stable and clinically meaningful predictors (**Figure 3C**, **Supplementary Table 1**): postoperative day 3 D-dimer (D-Dimer-D3), preoperative D-dimer (Pre-D-Dimer), percentage of neutrophil extracellular traps (NET%), carbohydrate antigen 19-9 (CA19-9), age (Age), portal vein resection type (PVR), and operative duration (Operation duration). These variables were subsequently used for model construction and validation.

**Figure 4:**
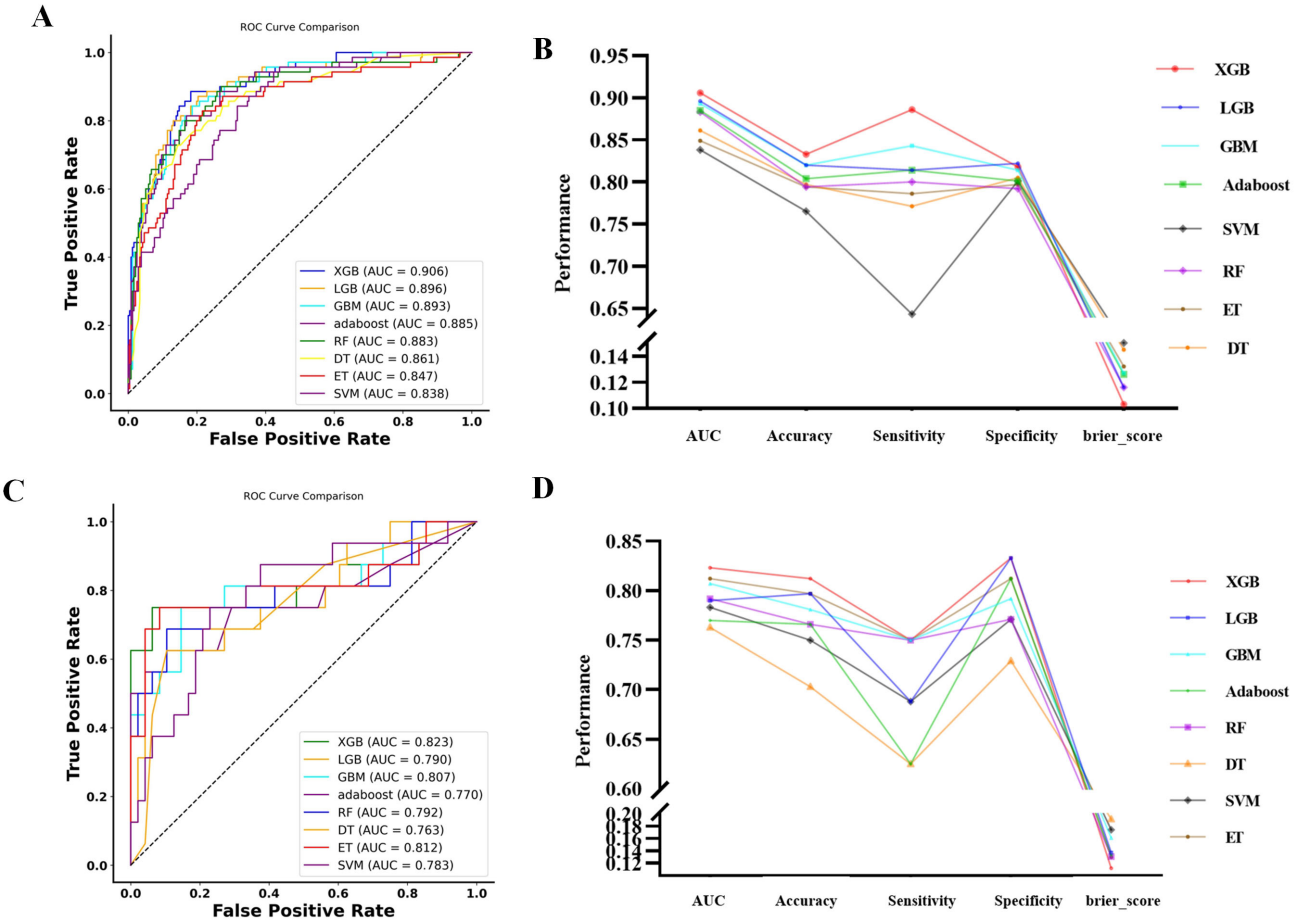
Performance evaluation of machine-learning models. **(A, B)** Receiver operating characteristic (ROC) curves and corresponding line plots of sensitivity, specificity, accuracy, and Brier score for the top eight machine-learning algorithms with the highest AUCs in the retrospective cohort. **(C, D)** Performance of the same eight algorithms in the prospective validation cohort, demonstrating the generalizability of model predictions.

Based on the selected features, 11 machine-learning algorithms were developed. In the retrospective cohort, model performance was comprehensively evaluated. Among all algorithms, the XGBoost model demonstrated the most favorable overall performance, achieving an AUC of 0.906, sensitivity of 0.886, specificity of 0.818, accuracy of 0.833, F1 score of 0.709, and Brier score of 0.103, significantly outperforming other models. The top eight algorithms ranked by AUC were visualized for comparison across multiple metrics (**Figures 4A, B**).

Consistent findings were observed in the prospective validation cohort (**Figures 4C, D**), confirming the robustness and generalizability of the XGBoost model. Detailed performance metrics for all 11 models in both cohorts are provided in **Supplementary Tables 2**, **3**, and the corresponding hyperparameter settings are summarized in **Supplementary Table 4**. Accordingly, the XGBoost model was selected as the optimal predictive algorithm for subsequent model development and risk-stratification analysis in this study.

#### 3.1.4 Model interpretability & SHAP-based feature analysis

Clinicians are often reluctant to adopt predictive models that lack intuitive interpretability, the SHapley Additive exPlanations (SHAP) method was employed to elucidate the output of the final model by quantifying the contribution of each variable to individual predictions. This interpretable framework provides two complementary forms of explanation: a global interpretation at the feature level and a local interpretation at the individual level.

**Figure 5:**
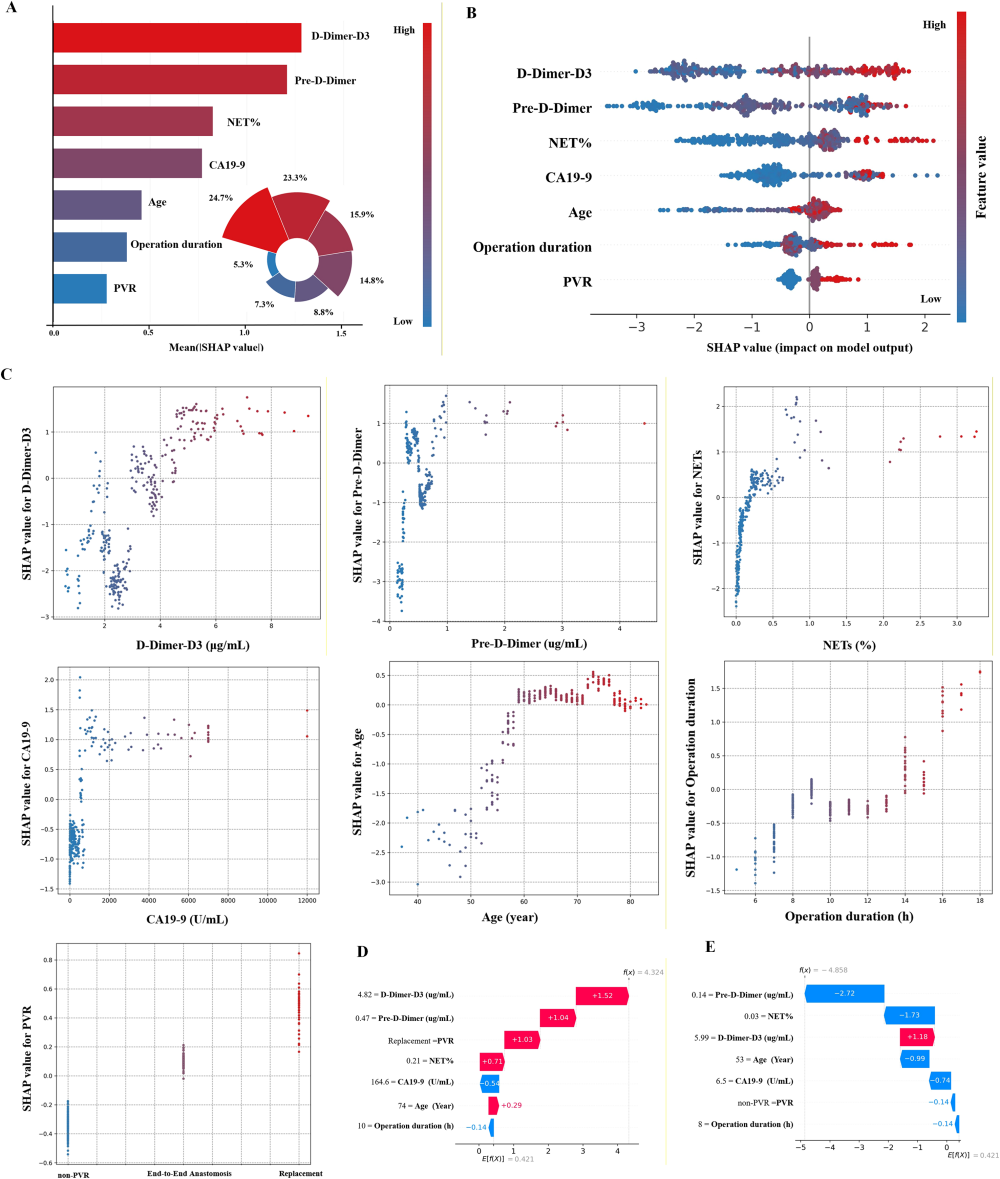
Global and local model interpretation using the SHAP method. **(A)** SHAP summary bar plot showing the mean absolute SHAP values of all features ranked by importance. **(B)** SHAP summary dot plot. The probability of postoperative PVT increases with the SHAP value of each feature; the color gradient represents actual feature values, where red indicates higher and blue indicates lower values. **(C)** SHAP dependence plots demonstrating how individual feature values influence model outputs; each dot represents one patient. **(D, E)** Waterfall plots illustrating the contribution of each feature to individual-level predictions. **(D)** represents a representative patient predicted as “PVT,” where positive SHAP values increase thrombotic risk. **(E)** represents a representative patient predicted as “non-PVT,” where cumulative feature effects favor a non-thrombotic outcome.

The global interpretation describes the overall behavior of the model. As shown in the SHAP summary plots (**Figures 5A, B**), the contribution of each feature to the model’s prediction was assessed using the mean absolute SHAP value, and features were ranked in descending order of importance. In addition, SHAP dependence plots illustrate how variations in individual features influence the model’s predicted outcomes. The relationships between the actual feature values and their corresponding SHAP values for all seven predictors are displayed in **Figure 5C**, where positive SHAP values indicate a higher predicted probability of PVT occurrence (i.e., a higher thrombotic risk).

The local interpretation, in contrast, demonstrates how personalized input data contribute to a specific prediction for an individual patient. **Figure 5D** depicts a representative case of a patient who developed postoperative PVT. The waterfall plot visualizes the actual measured values of each feature and their additive influence on the final prediction. As shown, elevated D-Dimer-D3, Pre-D-Dimer, PVR (Replacement), NET%, and Age values collectively shifted the prediction toward the “PVT” category, whereas CA19–9 and Operation duration exerted minimal or opposite effects. **Figure 5E** illustrates a contrasting example classified as “non-PVT”, in which the net effect of all feature contributions favored the non-thrombotic outcome.

The patient-level SHAP summary plot for the external validation cohort is presented in **Supplementary Figure 1**. In this plot, the x-axis represents individual patients, and the y-axis denotes the relative contribution of each feature. The red segments extending to the right indicate features that increased the likelihood of a “PVT” prediction, whereas blue segments denote those that favored a “non-PVT” decision. **Supplementary Figure 1** illustrates the explanatory plot of patients in the retrospective cohort. The x-axis represents individual patients, while the y-axis indicates the feature contribution. For each patient, the red portion reflects a greater likelihood of the model predicting a “PVT” outcome.

#### 3.1.5 Clinical implementation of a web-based application

**Figure 6:**
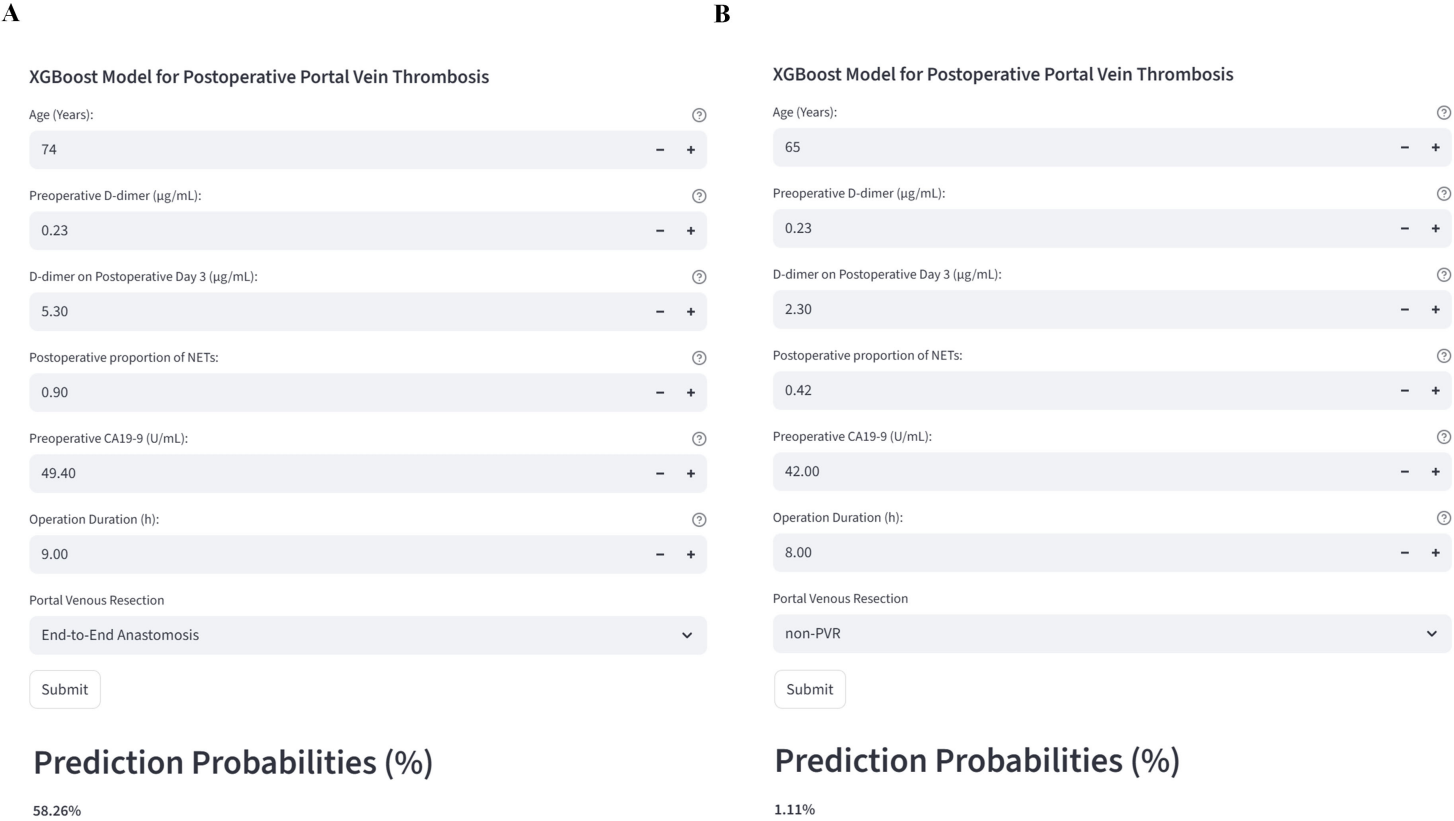
Deployment of the web-based prediction tool. The web application integrates the final XGBoost model incorporating seven predictive features for individualized PVT risk assessment. **(A, B)** Predicted postoperative PVT probabilities for two representative patients generated via the online platform.

Final predictive model was subsequently deployed as an online, publicly accessible web application to facilitate its dissemination and practical implementation in clinical settings (**Figure 6**). To further ensure the robustness of our final model, we conducted collinearity diagnostics to confirm that no redundant variables were retained. Variance inflation factor (VIF) analysis showed the following values: operation time 1.37, D-Dimer-D 1.07, Pre-D-Dimer 1.02, age 1.02, CA199 1.00, and NET% 1.02 - all below 5. We also constructed a correlation matrix among variables, which revealed only weak correlations (see **Supplementary Figure 2**). The primary purpose of this web-based tool is to provide clinicians with a simple, intuitive, and interactive platform for individualized risk assessment. By entering the actual values of the seven key predictive variables into the web interface, users can automatically trigger the embedded machine-learning algorithm, which then generates the real-time predicted probability of postoperative PVT for that specific patient. To demonstrate the clinical applicability of this tool, two representative patient cases were input into the system, and their corresponding postoperative PVT risk probabilities were computed and visualized (**Figures 6A, B**). Through this interactive and user-friendly interface, clinicians can rapidly obtain personalized, quantitative risk estimates, thereby enabling more informed postoperative management and decision-making. This tool substantially enhances the clinical operability and translational value of the developed model. The web application is freely available online at: https://prediction-model-for-pvt.streamlit.app/.

### 3.2 Prospective study

**Figure 7:**
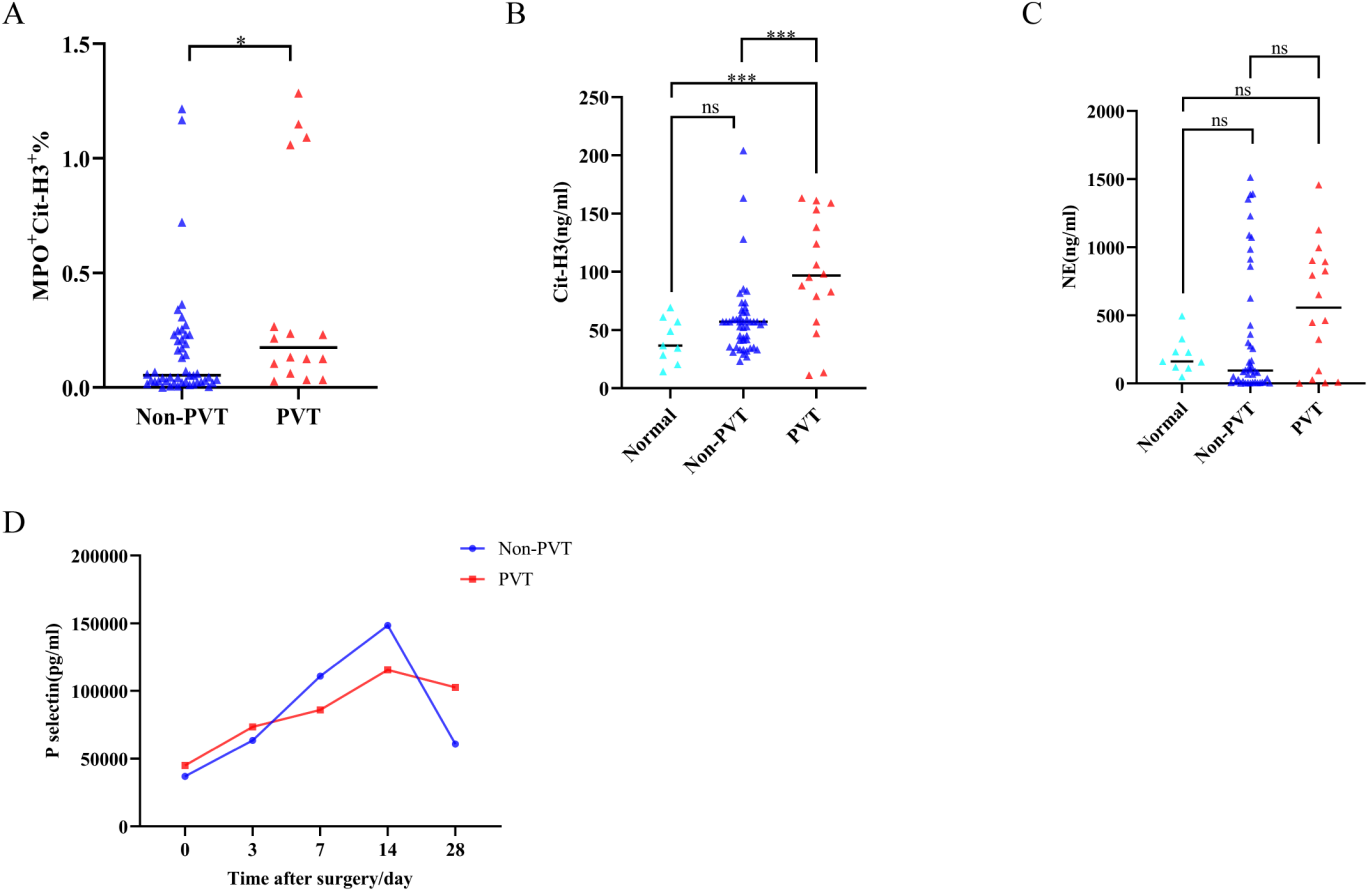
Expression of perioperative biomarkers in PDAC patients in this prospective study **(A–C)** The preoperative expression levels of MPO+Cit-H3+%, Cit-H3, and NE. **(D)** Dynamic expression of P-selectin during the perioperative period, * indicates P<0.05, *** indicates P<0.001.

In the prospective study, a total of 64 patients were included, of which 16 had PVT, with an incidence rate of 25.0%. Group all patients based on whether PVT occurred at one year postoperatively. MPO+Cit-H3+%, Cit-H3 levels in the PVT group were significantly higher than those in the non-PVT group (P<0.05), while NE did not show statistically significant differences between the two groups (**Figures 7A, C**). We also observed the dynamic changes of P-selectin during the perioperative period. Postoperative P-selectin levels in both groups gradually increased, peaking on day 14 and then declining. On postoperative days 7 and 14, the non-PVT group had higher levels than the PVT group, while on day 28, the PVT group had higher levels than the non-PVT group, but these differences were not statistically significant (**Figure 7D**).

## 4 Discussion

In this study, we investigated the relationship between NETs and PVT in patients undergoing surgery for PDAC. Our findings revealed a significant association between elevated NETs levels and the occurrence of postoperative PVT. These results suggest that NETs may serve as a potential biomarker for identifying PDAC patients at higher risk of postoperative thrombotic complications. Importantly, the observed associations were consistent across both retrospective and prospective cohorts, reinforcing the robustness of the findings and highlighting the clinical relevance of NETs in postoperative risk stratification. It should be noted that NET% was included in the model as a biological indicator of neutrophil activation and NET formation, not as causal evidence for thrombosis.

PVT is a complex pathophysiological process that may be caused by various diseases such as malignant tumors, local or systemic inflammatory diseases, and liver cirrhosis (23). For PDAC, especially locally advanced and borderline resectable PDAC, on one hand, highly malignant tumors activate the coagulation system, leading to a hypercoagulable state. On the other hand, advanced tumors often compress the PVS, causing blood stasis and frequently necessitating resection and reconstruction of the PVS, which greatly increases the likelihood of PVT (10, 11, 24). The risk factors for VTE primarily include patient-related factors, cancer-related factors, treatment-related factors (25). The results of this study indicate that the top-ranked factors identified by the XGBoost model were pre-D-dimer, postoperative day 3 D-dimer, NETs, age, surgical approach (vascular replacement > end-to-end anastomosis > non-PVR), CA19-9, and operation duration. D-dimer is a small protein fragment generated from the breakdown of cross-linked fibrin, reflecting the body’s fibrinolytic activity. Its elevated levels are clinically valuable for aiding in the diagnosis of VTE and other coagulation disorders (26). Due to the specific nature of pancreatic cancer surgery, resection and reconstruction of the portal venous system are often involved, which is also closely linked to the occurrence of postoperative PVT (11). Factors such as age, CA19–9 level, operation duration have been occasionally identified in other studies and are consistently associated with an established risk of PVT (27). Additionally, diabetes, JAK2V617F mutation, blood loss, inherited antithrombin (AT) deficiency, protein C (PC) deficiency, and protein S (PS) deficiency are also common risk factors for PVT (11, 28). Effectively assessing the risk of PVT based on independent risk factors and formulating individualized prevention strategies will help clinicians promptly identify people at high risk of PVT, take effective preventive measures, reduce the occurrence of complications, and prolong patients’ lives.

Currently, the relationship between PVT and NETs is still under-researched. Although evidence suggests that NETs play a significant role in the mechanism of various thrombus formations, their specific relationship with PVT has not been fully explored and understood. In this study, high NETs was found to be an independent risk factor for the occurrence of PVT after PDAC surgery. Compared to healthy individuals, tumor patients have more NETs formation in their neutrophils, which significantly increases the levels of thrombin and fibrin in peripheral blood (29). In a prospective study involving 946 patients with malignant tumors, the results showed that patients with high levels of Cit-H3 had a significantly higher cumulative incidence of VTE compared to those with low levels of Cit-H3 (30). In NETs, cell-free DNA (cfDNA) can form trimers with plasmin and fibrin, thereby hindering plasmin-mediated fibrin degradation, leading to more stable thrombi (31). Cit-H3 can induce the expression of tissue factor (TF) in endothelial cells, macrophages, and monocytes, thereby activating the extrinsic coagulation pathway and promoting thrombosis formation (32). It can also activate platelets through TLR2 and TLR4, promoting thrombin generation and leading to thrombosis (33). When analyzing thrombus tissue obtained from surgeries or autopsies, researchers used antibodies such as CD11b and Cit-H3 to label neutrophils and NETs. The results revealed the presence of neutrophils and NETs in the thrombus tissue of VTE patients (34). In a dextran sulfate sodium (DSS)-induced mouse model of inflammatory bowel disease (IBD), the administration of DNase I to degrade NETs significantly reversed the prothrombotic phenotype. Specifically, DNase I injection not only markedly reduced NETs content within the thrombi but, more critically, also restored key thrombosis parameters—including vessel occlusion time, thrombus weight, and thrombus length-to near-normal levels (35). In another study, researchers found that NETs formation was accompanied by high Tissue Factor (TF) expression, indicating their procoagulant potential. In functional inhibition assays, antibodies targeting DNA (the structural backbone of NETs) and TF independently suppressed their respective targets. Degrading NETs with DNase I reduced thrombin generation by approximately 40%, while a combination of DNase I and an anti-TF antibody led to a nearly 69% reduction (36). Further exploration is needed to understand the mechanisms linking NETs with PVT. Clarifying the specific role of NETs in PVT and their potential regulatory mechanisms could provide new therapeutic targets for clinical practice, and reduce the occurrence of related complications.

It is believed that NETs play a dual regulatory role in tumor prognosis by both promoting tumor growth, dissemination, and metastasis, and inhibiting tumor proliferation and invasion, akin to the dual anti-tumor and pro-tumor functions of neutrophils within tumors. In the tumor microenvironment (TME), tumor-associated neutrophils (TANs) can be classified into two phenotypes based on their functions: N1 (anti-tumor) phenotype and N2 (pro-tumor) phenotype (37). N1 phenotype neutrophils exhibit strong anti-tumor properties in various ways, including antibody-dependent and direct cytotoxic mechanisms, making them a powerful component of tumor defense (38). In contrast, N2 phenotype neutrophils promote tumor proliferation and metastasis by secreting a range of substances such as vascular endothelial growth factor (VEGF) and matrix metallopeptidase 9 (MMP-9), which stimulate tumor angiogenesis (39). The mechanisms by which NETs promote and inhibit tumors in tumor biology are still not fully understood. On the one hand, NETs can inhibit tumor cell migration, and co-culture with melanoma cells shows significant cytotoxic effects on melanoma cells, demonstrating a clear anti-tumor effect (40). On the other hand, NETs promote tumor cell proliferation by inhibiting apoptosis and can capture and adhere to circulating tumor cells (CTCs) in peripheral blood, leading to tumor cell metastasis (41–43). The components of NETs, such as NE, cathepsin G, and MMP-9, also play important roles in promoting tumorigenesis and progression. In a mouse lung cancer model, physiological concentrations of NE could directly induce tumor cell proliferation, while the use of NE inhibitors reduced lung tumor growth threefold (44). In NE-KO mice, the retention rate of tumor cells in the pulmonary circulation was significantly reduced after tail vein injection, indicating that the probability of tumor cells forming lung metastases in the circulation of NE-KO mice was significantly lower (45). In the Transwell cell invasion assay, cathepsin G significantly promoted the invasive capacity of hepatocellular carcinoma (HCC) cells *in vitro*; the expression of cathepsin G in HCC tissues was closely associated with the long-term prognosis of patients (46). MMP-9 had the ability to degrade components of the extracellular matrix (ECM) and release membrane-bound growth factors to establish a microenvironment conducive to tumor formation (47).

Recent studies have shown that specific markers of NETs, such as Cit-H3 and MPO in peripheral blood, can predict VTE events in patients with malignant tumors, novel coronavirus infection, and antiphospholipid syndrome (30, 48, 49). This may be due to various factors leading to neutrophil activation, resulting in the production of NETs and the release of various cytokines, which in turn promotes thrombosis formation. *In vitro* cell experiments, when the pancreatic cancer cell line AsPC-1 was co-cultured with neutrophils, it significantly increased the expression of NETs in neutrophils through soluble mediators; when AsPC-1 cells were co-cultured with platelets, they induced platelet activation, and the activated platelets could also rapidly stimulate neutrophils to produce NETs (50). In a mouse model, the number of neutrophils in the peripheral blood of nude mice carrying human pancreatic tumor BxPc-3 was significantly increased compared to the control group mice without tumors; compared to the control group mice, the biomarkers of activated neutrophils and NETs, such as NE and Cit-H3, were significantly elevated in the plasma of tumor-bearing mice; in the inferior vena cava stasis model of mice, the levels of Ly6G and Cit-H3 in the thrombi of tumor-bearing mice were significantly increased (51). Conducting a thorough evaluation of relevant biomarkers can aid clinicians in identifying high-risk populations for PVT, thereby enabling the development of personalized treatment strategies to enhance patient prognosis.

This study also has some limitations. Firstly, this is a single-center study with a relatively small sample size, which may subject the results to selection bias and sample size limitations. Further large-sample multi-center studies are needed to improve the generalizability and credibility of the findings. Secondly, the number of biomarkers included in this study is limited, which may result in missing many effective biomarkers. In the future, it is necessary to screen effective biomarkers through high-throughput screening techniques and conduct perioperative monitoring at multiple time points. This will provide more substantial and powerful evidence for the prevention and treatment of PVT. Finally, the incidence of PVT in patients with PDAC gradually increases over time, which is closely related to tumor recurrence. Therefore, future prospective studies should extend the follow-up period to obtain more reliable research results.

## 5 Conclusion

Our study demonstrates that NETs are significantly associated with the occurrence of PVT in patients with PDAC following surgery. By assessing NETs-related biomarkers such as Cit-H3 and dynamic D-dimer levels, clinicians may be able to identify high-risk patients early and implement targeted preventive strategies. This approach holds promise for improving individualized postoperative management and ultimately enhancing the long-term outcomes of PDAC patients.

## Data Availability

The original contributions presented in the study are included in the article/**Supplementary Material**. Further inquiries can be directed to the corresponding authors.

## Supplementary material

Supplementary Figure 1 Local model explanation using the SHAP method in the internal validation cohort. Each patient is represented along the x-axis, while the y-axis denotes the contribution of individual features. Red segments extending to the right indicate features that increase the likelihood of a “PVT” prediction, whereas blue segments correspond to features contributing to a “non-PVT” outcome.

Supplementary Figure 2 Correlation of variables in the XGB model. Blue indicates a positive correlation between variables, while red indicates a negative correlation. The darker the color, the stronger the correlation. The correlation coefficients are displayed in the figure.

Supplementary Table 1 Variables selected by the LASSO regression and RFE methods.

Supplementary Table 2 Performance metrics of the eleven machine-learning algorithms in the retrospective cohort.

Supplementary Table 3 Performance metrics of the eleven machine-learning algorithms in the prospective validation cohort.

Supplementary Table 4 Hyperparameter settings of the eleven machine-learning algorithms.
