# Supplementary figures and images for: Organ-specific role of neutrophil extracellular traps in shaping the postoperative thrombo-inflammatory niche in pancreatic ductal adenocarcinoma: a multi-model machine learning and mechanistic study
Source: Front Immunol. 2025 Dec 9;16:1728391. doi: 10.3389/fimmu.2025.1728391 (PMC12722873; doi:10.3389/fimmu.2025.1728391)

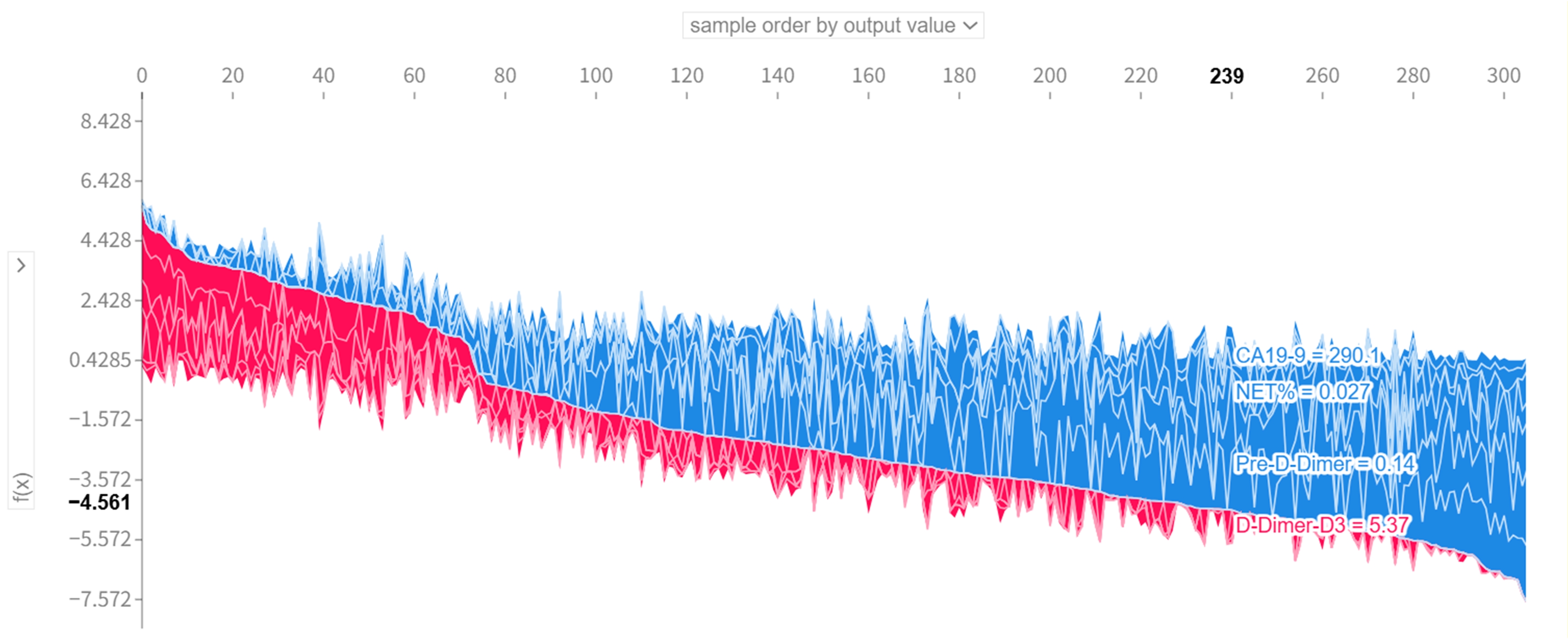

Supplement: Supplementary Figure 1 — Local model explanation using the SHAP method in the internal validation cohort. Each patient is represented along the x-axis, while the y-axis denotes the contribution of individual features. Red segments extending to the right indicate features that increase the likelihood of a “PVT” prediction, whereas blue segments correspond to features contributing to a “non-PVT” outcome. [file Image1.jpg]

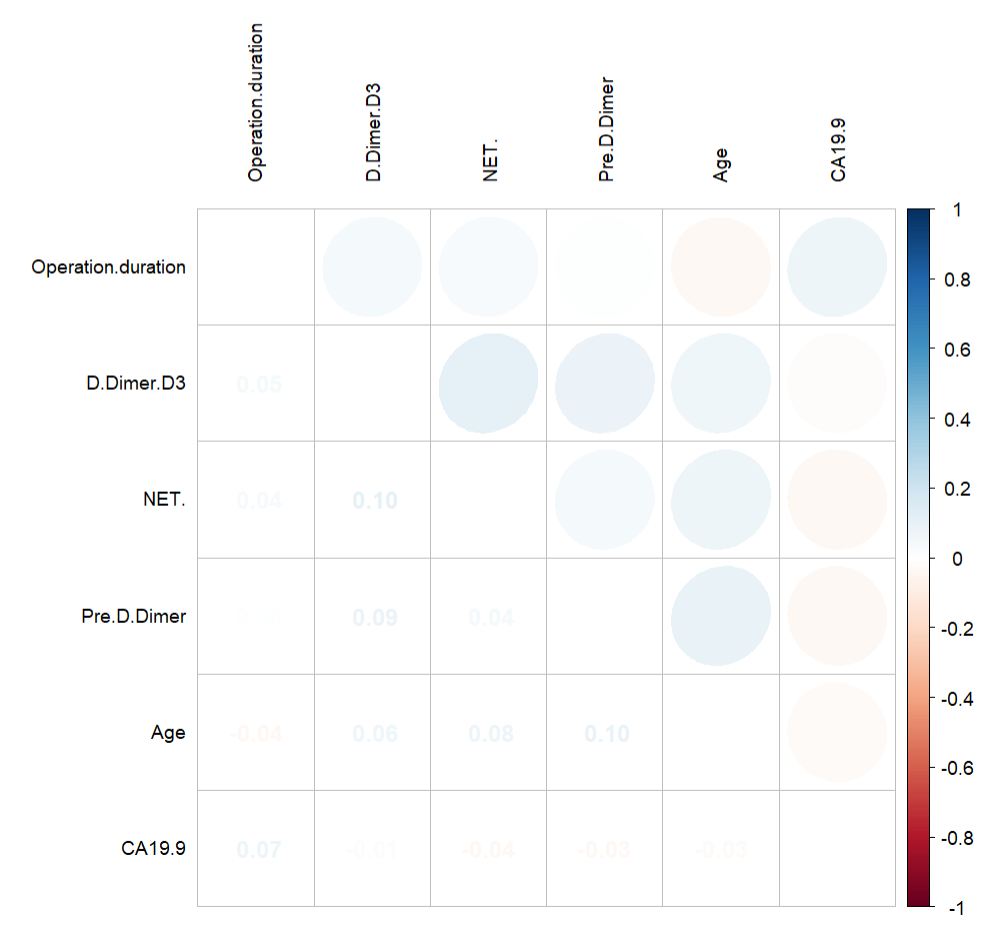

Supplement: Supplementary Figure 2 — Correlation of variables in the XGB model. Blue indicates a positive correlation between variables, while red indicates a negative correlation. The darker the color, the stronger the correlation. The correlation coefficients are displayed in the figure. [file Image2.tif]
