# Supplementary material for: Organ-specific role of neutrophil extracellular traps in shaping the postoperative thrombo-inflammatory niche in pancreatic ductal adenocarcinoma: a multi-model machine learning and mechanistic study
Source: Front Immunol. 2025 Dec 9;16:1728391. doi: 10.3389/fimmu.2025.1728391 (PMC12722873; doi:10.3389/fimmu.2025.1728391)
Supplement: Supplementary Table 1 — Variables selected by the LASSO regression and RFE methods. [file Table1.docx]

| Variables | Training set | | P Value | Test set | | P Value |
| --- | --- | --- | --- | --- | --- | --- |
|  | Non-PVT  (n=236) | PVT  (n=70) |  | Non-PVT  (n=48) | PVT  (n=16) |  |
| Age (y)^b^ | 64.00 (57.00, 71.00) | 66.00 (63.00, 71.75) | 0.032 | 59.50 (55.00, 66.00) | 66.50 (63.50, 69.25) | 0.015 |
| Female sex^a^ | 99 (41.95%) | 29 (41.43%) | 0.989 | 26 (54.17%) | 6 (37.50%) | 0.386 |
| Underlying comorbidity^a^ |  |  |  |  |  |  |
| Hypertension | 98 (41.53%) | 20 (28.57%) | 0.069 | 19 (39.58%) | 6 (37.50%) | 0.978 |
| DM | 73 (30.93%) | 27 (38.57%) | 0.293 | 16 (33.33%) | 3 (18.75%) | 0.353 |
| CHD | 30 (12.71%) | 5 (7.14%) | 0.284 | 9 (18.75%) | 2 (12.50%) | 0.716 |
| History of abdominal  Smoking | 13 (5.51%)  77 (32.63%) | 5 (7.14%)  28 (40.00%) | 0.318 | 3 (6.25%)  12 (25.00%) | 1 (6.25%)  2 (12.50%) | 0.999  0.487 |
| Surgical approach^a^ |  |  | <0.001 |  |  | 0.005 |
| Non-PVR | 138 (17.28%) | 24 (16.90%) |  | 33 (68.75%) | 5 (31.25%) |  |
| End-to-end anastomosis | 78 (32.30%) | 27 (25.35%) |  | 13 (27.08%) | 6 (37.50%) |  |
| Vascular replacement | 20 (14.61%) | 19 (21.13%) |  | 2 2 (4.17%) | 5 (31.25%) |  |
| Operation time (h)^b^ | 9.00 (8.00, 12.00) | 10.00 (9.00, 13.00) | 0.005 | 9.00 (8.00, 12.00) | 10.00 (9.00, 11.25) | 0.229 |
| Hemorrhage (mL)^b^ | 500.00 (400.00, 800.00) | 600.00 (400.00, 975.00) | 0.018 | 500.00 (400.00, 800.00) | 550.00 (400.00, 800.00) | 0.383 |
| Transfusion volume (ml)^a^  0  ≥400  ≥800 | 131 (55.51%)  44 (18.64%)  61 (25.85%) | 27 (38.57%)  16 (22.86%)  27 (38.57%) | 0.038 | 34 (70.83%)  5 (10.42%)  9 (18.75%) | 9 (56.25%)  5 (31.25%)  2 (12.50%) | 0.155 |
| NETs (%)^b^ | 0.08 (0.04, 0.23) | 0.25 (0.14, 0.55) | <0.001 | 0.13 (0.08, 0.20) | 0.23 (0.06, 0.33) | 0.195 |
| TNM^a^ |  |  | 0.061 |  |  | 0.309 |
| I | 65 (27.54%) | 10 (14.29%) |  | 12 (25.00%) | 2 (12.50%) |  |
| II | 108 (45.76%) | 35 (50.00%) |  | 25 (52.08%) | 7 (43.75%) |  |
| III | 63 (26.69%) | 25 (35.71%) |  | 11 (22.92%) | 7 (43.75%) |  |
| Differentiation^a^ |  |  | 0.077 |  |  | 0.526 |
| Poorly | 71 (30.08%) | 30 (42.86%) |  | 16 (33.33%) | 4 (25.00%) |  |
| Moderately | 146 (61.86%) | 38 (54.29%) |  | 29 (60.42%) | 12 (75.00%) |  |
| Highly | 19 (8.05%) | 2 (2.86%) |  | 3 (6.25%) | 0 (0.00%) |  |
| Chemotherapy, only^a^ | 138 (58.47%) | 35 (50.00%) | 0.263 | 28 (58.33%) | 12 (75.00%) | 0.372 |
| Laboratory values^b^ |  |  |  |  |  |  |
| WBC (x 10⁹/L) | 5.75 (4.60, 6.90) | 5.85 (4.90, 7.57) | 0.319 | 5.85 (4.77, 6.67) | 5.40 (4.40, 6.45) | 0.558 |
| Neutrophil (x 10⁹/L) | 3.60 (2.70, 4.60) | 3.70 (3.00, 4.97) | 0.151 | 3.51 (2.88, 4.36) | 3.64 (2.99, 4.36) | 0.648 |
| PLT (x 10⁹/L) | 204.00 (165.00, 255.25) | 217.00 (170.50, 268.00) | 0.170 | 199.00 (153.75, 246.75) | 185.50 (146.25, 277.75) | 0.936 |
| Hemoglobin (g/dL) | 124.00 (113.00, 134.00) | 119.50 (111.25, 137.50) | 0.859 | 120.50 (113.00, 139.25) | 129.50 (114.75, 139.00) | 0.679 |
| Albumin (g /L) | 37.85 (34.15, 40.82) | 38.05 (34.47, 42.35) | 0.515 | 36.85 (32.90, 39.90) | 38.35 (32.68, 40.02) | 0.792 |
| Total bilirubin (umol /L) | 19.55 (10.20, 123.53) | 36.80 (11.22, 149.25) | 0.287 | 23.95 (11.52, 101.70) | 16.45 (12.60, 78.50) | 0.664 |
| Pre-D-dimer (ug/mL) | 0.41 (0.25, 0.57) | 0.52 (0.39, 0.76) | 0.039 | 0.37 (0.23, 0.53) | 0.66 (0.50, 0.91) | <0.001 |
| D-dimer(D3) (ug/mL) | 2.51 (2.08, 3.76) | 3.97 (3.12, 4.84) | <0.001 | 2.40 (1.81, 3.11) | 3.95 (2.53, 4.82) | 0.006 |
| ALT ((U/L) | 32.00 (16.00, 92.50) | 48.00 (20.25, 117.00) | 0.106 | 31.00 (15.75, 60.25) | 49.00 (15.00, 137.50) | 0.281 |
| AST ((U/L) | 31.00 (18.00, 65.00) | 41.50 (21.25, 97.50) | 0.041 | 23.50 (16.00, 49.50) | 56.50 (24.75, 102.00) | 0.057 |
| GGT (U/L) | 107.00 (21.00, 430.50) | 199.00 (29.25, 472.50) | 0.139 | 135.00 (17.75, 299.75) | 31.00 (18.50, 183.00) | 0.721 |
| CA19-9 (U /mL) | 175.05 (38.90, 494.15) | 420.45 (40.58, 1297.18) | 0.011 | 87.50 (9.40, 280.00) | 466.15 (148.33, 1676.60) | 0.003 |
| CEA (ng /mL) | 2.10 (1.30, 4.15) | 3.10 (1.65, 4.97) | 0.078 | 3.15 (1.90, 4.32) | 4.75 (3.25, 7.43) | 0.052 |

**Table 1. Clinicopathologic Features and Operative Characteristics**

PVT, Portal vein thrombosis; DM, Diabetes Mellitus; CHD, Coronary Heart Disease; PVR, portal venous resection; NETs, neutrophil extracellular traps; WBC,White Blood Cell; PLT, Platelet Count; ALT, Alanine aminotransferase; AST, Aspartate aminotransferase; GGT, Gamma-glutamyl transferase; CA19-9, Carbohydrate antigen 19-9; CEA, Carcinoembryonic antigen. ^a^ Number (%); ^b^ Median (range).

**Supplementary Table 1. Variables selected by the LASSO regression and RFE methods**

| Lasso Regression​​ | | Recursive Feature Elimination​​ | |
| --- | --- | --- | --- |
| Features | Coefficient | Features | Importance |
| NET% | 1.185168 | D-Dimer-D3 | 0.35639597 |
| PVR2 | 1.104261 | NET% | 0.20188807 |
| Diabetes | 0.401107 | Pre-D-Dimer | 0.16337178 |
| Pre-D-Dimer | 0.385696 | 'Age' | 0.10581373 |
| Smoking | 0.299206 | CA19-9 | 0.07851551 |
| D-Dimer-D3 | 0.166066 | Operation duration | 0.03159356 |
| Differentiation2 | 0.126063 | PVR | 0.02535182 |
| TNM2 | 0.046601 | CEA | 0.0190998 |
| Age | 0.023231 | AST | 0.01796975 |
| Operation duration | 0.013972 | - | - |
| Alb | 0.005325 | - | - |
| AST | 0.002339 | - | - |
| PLT | 0.001822 | - | - |
| Bleeding volume | 0.000276 | - | - |
| CA19-9 | 9.55E-05 | - | - |
| Hypertension | -0.51031 | - | - |

**Supplementary Table 2. Performance metrics of the eleven machine-learning algorithms in the retrospective cohort**

| Models | AUC | Sensitivity | Specificity | PPV | NPV | Accuracy | F1 score | Brier score |
| --- | --- | --- | --- | --- | --- | --- | --- | --- |
| XGB | 0.906 | 0.886 | 0.818 | 0.590 | 0.960 | 0.833 | 0.709 | 0.103 |
| lightGBM | 0.896 | 0.814 | 0.822 | 0.576 | 0.937 | 0.820 | 0.675 | 0.116 |
| GBM | 0.893 | 0.843 | 0.814 | 0.573 | 0.946 | 0.820 | 0.682 | 0.127 |
| AdaBoost | 0.885 | 0.814 | 0.801 | 0.548 | 0.936 | 0.804 | 0.655 | 0.126 |
| RF | 0.883 | 0.800 | 0.792 | 0.533 | 0.930 | 0.794 | 0.640 | 0.116 |
| DT | 0.861 | 0.771 | 0.805 | 0.540 | 0.922 | 0.797 | 0.635 | 0.145 |
| ET | 0.847 | 0.786 | 0.797 | 0.534 | 0.926 | 0.794 | 0.636 | 0.132 |
| SVM | 0.838 | 0.643 | 0.801 | 0.489 | 0.883 | 0.765 | 0.556 | 0.150 |
| LR | 0.824 | 0.750 | 0.688 | 0.444 | 0.892 | 0.703 | 0.558 | 0.152 |
| ANN | 0.810 | 0.625 | 0.812 | 0.526 | 0.867 | 0.766 | 0.571 | 0.110 |
| KNN | 0.802 | 0.688 | 0.792 | 0.524 | 0.884 | 0.766 | 0.595 | 0.136 |

**Supplementary Table 3. Performance metrics of the eleven machine-learning algorithms in the prospective validation cohort**

| Models | AUC | Sensitivity | Specificity | PPV | NPV | Accuracy | F1 score | Brier score |
| --- | --- | --- | --- | --- | --- | --- | --- | --- |
| XGB | 0.823 | 0.750 | 0.833 | 0.600 | 0.909 | 0.812 | 0.667 | 0.112 |
| lightGBM | 0.790 | 0.688 | 0.833 | 0.579 | 0.889 | 0.797 | 0.629 | 0.137 |
| GBM | 0.807 | 0.750 | 0.792 | 0.545 | 0.905 | 0.781 | 0.632 | 0.161 |
| AdaBoost | 0.770 | 0.625 | 0.812 | 0.526 | 0.867 | 0.766 | 0.571 | 0.133 |
| RF | 0.792 | 0.750 | 0.771 | 0.522 | 0.902 | 0.766 | 0.615385 | 0.131 |
| DT | 0.763 | 0.625 | 0.729 | 0.435 | 0.854 | 0.703 | 0.512821 | 0.191 |
| ET | 0.812 | 0.750 | 0.812 | 0.571 | 0.907 | 0.797 | 0.649 | 0.130 |
| SVM | 0.783 | 0.688 | 0.771 | 0.500 | 0.881 | 0.750 | 0.579 | 0.174 |
| LR | 0.781 | 0.814 | 0.610 | 0.383 | 0.917 | 0.657 | 0.521 | 0.189 |
| ANN | 0.740 | 0.600 | 0.826 | 0.506 | 0.874 | 0.775 | 0.549 | 0.172 |
| KNN | 0.798 | 0.743 | 0.737 | 0.456 | 0.906 | 0.739 | 0.565 | 0.139 |

**Supplementary Table 4. Hyperparameter settings of the eleven machine-learning algorithms**

| Models |  |  |  |  |  |
| --- | --- | --- | --- | --- | --- |
| XGB | n_estimators | learning_rate | subsample | scale_pos_weight | max_depth |
|  | 296 | 0.036 | 0.9 | 4.28 | 3 |
| lightGBM | n_estimators | max_depth | num_leaves | learning_rate |  |
|  | 126 | 7 | 97 | 0.03 |  |
| GBM | n_estimators | max_depth | learning_rate | min_samples_split |  |
|  | 130 | 8 | 0.1 | 6 |  |
| AdaBoost | n_estimators | learning_rate | max_depth | min_samples_split | min_samples_leaf |
|  | 201 | 0.24 | 5 | 5 | 10 |
| RF | n_estimators | max_depth | min_samples_split | max_features |  |
|  | 164 | 10 | 16 | Log2 |  |
| DT | criterion | max_depth | min_samples_split | min_samples_leaf |  |
|  | gini | 21 | 13 | 9 |  |
| ET | n_estimators | max_depth | min_samples_split | min_samples_leaf |  |
|  | 62 | 8 | 2 | 3 |  |
| SVM | C | kernel |  |  |  |
|  | 98 | rbf |  |  |  |
| LR | solver | max_iter |  |  |  |
|  | lbfgs | 53 |  |  |  |
| ANN | hidden_layer_sizes | activation | solver | batch_size | max_iter |
|  | (100,) | relu | sgd | 64 | 70 |
| KNN | n_neighbors | p | leaf_size |  |  |
|  | 15 | 1 | 35 |  |  |
